# Tracking the Progress of Biocomposites Based on Poly(3-hydroxybutyrate) with Hypromellose Additives via Thermal Analysis, Mechanical Properties, and Biological Studies

**DOI:** 10.3390/ijms27031596

**Published:** 2026-02-06

**Authors:** Karolina Maternia-Dudzik, Łukasz Ożóg, Zuzanna Bober, Rafał Oliwa, Mariusz Oleksy, Angelika Kamizela, Agnieszka Szyszkowska, Katarzyna Rafińska, Weronika Gonciarz, Kamil Gancarczyk, Anna Czerniecka-Kubicka

**Affiliations:** 1 Laboratory of Physical Chemistry and Biophysics, Centre for Innovative Research in Medical and Natural Sciences, Faculty of Medicine, University of Rzeszow, 35-959 Rzeszow, Poland; kmaternia@ur.edu.pl (K.M.-D.); lozog@ur.edu.pl (Ł.O.); zbober@ur.edu.pl (Z.B.); angelikakamizela@gmail.com (A.K.); 2 Department of Pharmaceutical Technology and Medical Physics, Faculty of Medicine, University of Rzeszow, 35-959 Rzeszow, Poland; 3 Department of Polymer Composites, Faculty of Chemistry, Rzeszow University of Technology, 35-959 Rzeszow, Poland; oliwa@prz.edu.pl (R.O.); molek@prz.edu.pl (M.O.); 4 Provincial Hospital in Kielce, 25-736 Kielce, Poland; szyszkowska.agnieszka@gmail.com; 5 Department of Environmental Chemistry and Bioanalytics, Faculty of Chemistry, Nicolaus Copernicus University in Torun, 87-100 Torun, Poland; katraf@umk.pl; 6 Department of Immunology and Infectious Biology, Institute of Microbiology, Biotechnology and Immunology, Faculty of Biology and Environmental Protection, University of Lodz, 90-237 Lodz, Poland; weronika.gonciarz@biol.uni.lodz.pl; 7 Department of Material Science, Faculty of Mechanical Engineering and Aeronautics, Rzeszow University of Technology, 35-959 Rzeszow, Poland; kamilgancarczyk@prz.edu.pl

**Keywords:** poly(3-hydroxybutyrate), hypromellose, biocomposite, biocompatible materials

## Abstract

Poly(3-hydroxybutyrate) (P3HB) was used to produce biocompatible composites with hypromellose as an additive. The study aimed to assess their biological and mechanical properties, as well as specific thermal parameters and phase content. Differential scanning calorimetry was applied to analyze the phase transitions of both biocomposites and the polymer matrix. Furthermore, the thermal parameters—encompassing both non-equilibrium and equilibrium states—of the biocomposites and unfilled P3HB were evaluated according to their thermal history. Using equilibrium parameters such as the heat of fusion for fully crystalline materials and the heat capacity change at the glass transition for fully amorphous composites, we estimated the degrees of crystallinity as well as the mobile and rigid amorphous fractions. Adding hypromellose to the P3HB matrix reduced crystallinity compared to the unfilled material. At the same time, an increase in the amorphous phase was observed. It was also discovered that the rigid amorphous fraction exists solely in biocomposites containing 2% by mass of filler. Thermogravimetric analysis showed that the thermal stability of all biocomposites surpasses that of unfilled P3HB. Adding an extra 1% filler by mass raises the degradation temperature by about 37 °C compared to unfilled P3HB. The immunosafety of the tested biocomposites, with very low or no endotoxin contamination, was confirmed in accordance with Food and Drug Administration and European Medicines Agency guidelines. The study clearly demonstrates the influence of the filler in the P3HB matrix on various structural, thermal, mechanical, and biological properties of the prepared biocomposites.

## 1. Introduction

Poly(3-hydroxybutyrate) (P3HB) belongs to the polyhydroxyalkanoates (PHA) family, which consists of biodegradable polyesters produced by microorganisms as intracellular storage compounds [1,2,3]. The chemical structure of PHAs is based on hydroxyacid monomer units, which allows extensive chemical modification and fine-tuning of their physicochemical and mechanical properties for specific biomedical and engineering applications [2,3,4]. As thermoplastics, PHAs can be processed using conventional methods, and their mechanical performance can be adjusted through copolymerization, blending with other polymers, or incorporation of nanomaterials [4]. PHAs undergo enzymatic degradation in the environment and within living organisms, yielding non-toxic metabolites; their biocompatibility [5,6], combined with the ability to be sterilized without loss of properties [1], makes them attractive candidates for biomedical applications [1,4,5,7]. The discovery of PHB in 1926 initiated a series of studies exploring its potential uses [8]. P3HB is the most extensively studied PHA due to its widespread availability and favorable physicochemical and biological properties, including non-cytotoxicity, biodegradability, biocompatibility, absence of mutagenicity, and thermoplasticity [9,10]. Since degradation products like 3-hydroxybutyrate are naturally found in human metabolism, they are less likely to trigger adverse immune responses [1,6].

P3HB can be produced from simple and renewable substrates, such as sugars, fats, plant biomass, or natural gas (CH_4_), using microorganisms including *Ralstonia eutropha*, *Cupriavidus necator*, and *Escherichia coli*. The feasibility of industrial-scale production makes P3HB an economically attractive material for biomedical applications [3,4,11,12,13]. P3HB exhibits thermoplastic properties comparable to conventional petrochemical-based polymers, enabling processing through standard techniques [14]. It is a semi-crystalline polyester with a high melting temperature of 173–180 °C and a glass transition temperature of approximately 0–5 °C, with crystallinity levels of 65–80%, resulting in high rigidity and mechanical properties comparable to isotactic polypropylene [15,16].

The use of P3HB is hindered by its intrinsic stiffness and brittleness, as well as by low thermal stability in the vicinity of its melting temperature. The combination of high crystallinity and the proximity of melting and degradation temperatures creates a narrow thermal processing window, representing a primary obstacle for unmodified P3HB [15]. Improving these characteristics could expand the potential applications of this material. The mechanical performance of P3HB can be enhanced by copolymerization, the addition of plasticizers, the incorporation of nanomaterials, or natural fibers. Such strategies improve strength, flexibility, and processability while enabling the design of materials with controlled biodegradation [4,5]. Furthermore, P3HB exhibits shape-memory properties [17], allowing it to partially recover its original form in response to external stimuli, such as temperature, which enhances its potential in medical applications, including implants and tissue engineering scaffolds [18,19,20].

One of the more widely described applications of P3HB is drug delivery systems (DDS). The combination of P3HB with gelatin or various modifications of P3HB is being investigated for possible use as drug carriers, e.g., 5-fluorouracil (FU) and methotrexate (MT), azathioprine (AZA) [21,22]. Particular attention is drawn to the possibility of using P3HB in tissue engineering. Currently, the gold standard of tissue reconstruction is autologous transplantation due to the lack of stimulation of the recipient’s immune response. Autologous grafts are great for the reconstruction of small bone defects; however, in the case of larger defects, it is necessary to use other reconstruction materials. It is crucial that the materials used activate the recipient’s immune system minimally, and thus do not cause transplant rejection. P3HB, as a biocompatible material, is an object of tissue engineering research. Currently, work is underway on P3HB-based materials inoculated with human osteoblasts [23] and mesenchymal stem cells [24], which give promising results. P3HB is also used in the treatment of bone defects associated with osteomyelitis. Modifications enabling the introduction of antibacterial substances at the site of the bone defect seem to be useful for this purpose [25].

One of the compounds that may be an interesting addition to P3HB-based implants is methylcellulose (HYP), also known as hypromellose. The substrate for its synthesis is cellulose, commonly found in plants. HYP belongs to the group of ethers obtained by substituting hydrogen atoms from cellulose hydroxyl groups for methyl and hydroxypropyl groups [26]. Solubility in water is a feature that distinguishes HYP from cellulose [27]. Cellulose has hydroxyl groups responsible for the formation of hydrogen bonds, enabling the formation of an ordered crystalline structure of this compound. These groups are replaced with methoxy groups, which contribute to changing the properties of HYP. HYP, as an easy-to-obtain, biocompatible, and biodegradable material, has been used in the production of hydrogels promoting the repair of joint cartilage [27] and as a drug carrier in the pharmaceutical industry [28]. Currently, research is underway into the use of HYP in the production of bioinks for the bioprinting process [26].

The aim of this paper is to examine the effect of hypromellose on the P3HB matrix and to evaluate the properties of the material in terms of applications in the biomedical industry. Mechanical tests, structural and thermal properties, and biocompatibility with a living organism have been established.

## 2. Results and Discussion

The biocomposites were produced by melt mixing P3HB with varying hypromellose contents—0.5, 1, 2, and 3 per cent by mass—in a co-rotating twin-screw micro-extruder. Before the extrusion, the filler was homogenized with the polymeric matrix using an electric grinder. The obtained biocomposites were labeled as follows: MP3HB-0.5, MP3HB-1, MP3HB-2, and MP3HB-3, respectively.

**Figure 1:**
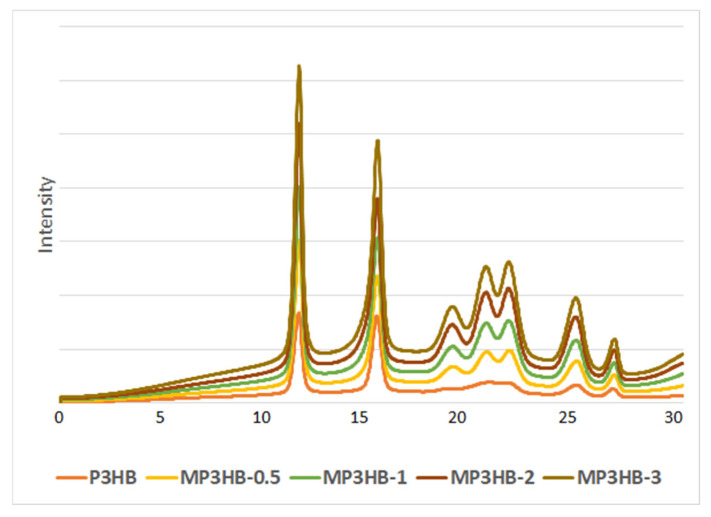
Diffractograms of the unfilled P3HB and its biocomposites with 0.5, 1, 2, and 3 wt.-% of hypromellose (designated MP3HB-0.5, MP3HB-1, MP3HB-2, and MP3HB-3, respectively).

Figure 1 displays diffractograms of unfilled P3HB and its biocomposites containing 0.5, 1, 2, and 3 wt.-% of hypromellose. The XRD analysis of P3HB and its composites obtained using hypromellose allowed us to observe the effect of introducing the additive on changing the phase composition. The most intense reflection, observed for all tested samples at 2θ ≈ 13–14°, is characteristic of the crystalline phase of P3HB and corresponds to the ordering of polymer chains in the dominant crystallographic plane, which is consistent with previously reported XRD patterns of crystalline P3HB [15,16,29]. The high intensity and relatively sharp shape of this peak confirm the semi-crystalline nature and high degree of crystallinity of the P3HB matrix. Increasing the modifier content in MP3HB composites leads to changes in the intensity of this reflection, which indicates the influence of the additive on the P3HB crystallization process, probably by affecting the crystallization kinetics and lamellar organization during cooling, despite increased segmental mobility in the amorphous phase observed by DSC. The lack of a clear shift in the peak maximum position suggests that the structure of the P3HB unit cell remains unchanged. A change in the shape of the peaks can be observed in the range of angle 2 theta from 18 to 24. The wide, indistinct peak on the P3HB diffractogram transformed into three sharp peaks on the diffractograms of the P3HB/hypromellose composites. Additional reflections in the range of 2θ ≈ 20–26° may indicate the presence of phases originating from the modifying component or changes in the order of the crystal structure of the matrix. The absence of significant shifts in peak positions suggests that the crystalline structure of P3HB is preserved, while the additive influences its organization and the proportion of the crystalline phase. The effect of raising the background was visible in the increase in the amount of hypromellose used. Therefore, it can be clearly stated that the introduction of hypromellose increased the amorphous phase content, and P3HB increases its degree of crystallinity in new materials. This can be observed by the increase in the intensity of the peaks.

According to the Scherrer equation, the crystallite size for the tested samples was calculated [30]:

(1) $$D\;=\;\frac{K\lambda }{Bcos\theta }$$

D—average crystallite size, nm;

*K*—shape constant, 0.9;

λ—0.154 nm;

*B*—FWHM. 

The FWHM measurement of the largest reflection in the diffraction patterns in the range 2θ = 13–14° was used to calculate the average crystallite size (Table 1).

**Figure 2:**
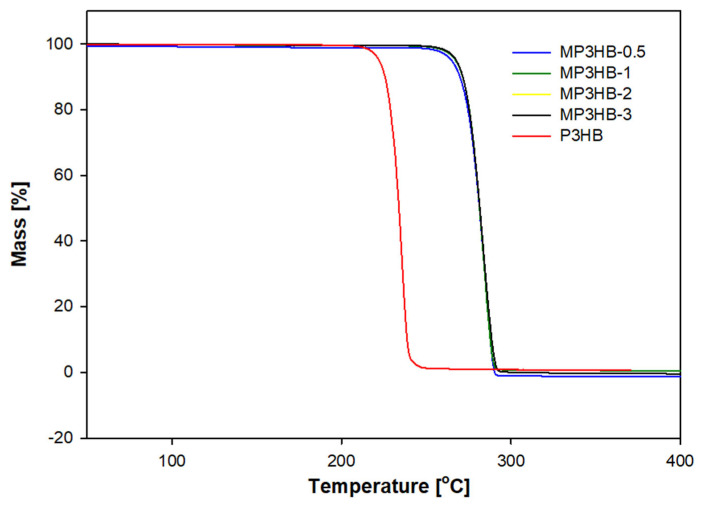
Thermogravimetric analysis was conducted on unfilled poly(3-hydroxybutyrate) and its biocomposites with 0.5, 1, 2, and 3 mass % hypromellose. These biocomposites are designated as MP3HB-0.5, MP3HB-1, MP3HB-2, and MP3HB-3, respectively.

The obtained results are very similar and fall within the literature data contained in works describing the measurement of crystallite size of P3HB materials, and are in the range of approximately 15–35 nm [29,31]. The limited variation in crystallite size suggests that hypromellose affects phase composition rather than crystal growth, indicating a predominantly homogeneous dispersion within the P3HB matrix. To assess the durability, processability, and functional performance of the biocomposites, thermogravimetric analysis (TGA) was conducted (Figure 2). The TG profiles revealed that all biocomposites possess enhanced thermal stability compared with neat P3HB. The addition of hypromellose to the P3HB matrix resulted in an increase in the degradation temperature, with a 2% weight loss observed at a higher temperature of 222.8 °C relative to the unfilled polymer. The obtained biocomposites were reported in the same conditions, 245.6, 260.1, 250.2, and 249.6 °C in the case of MP3HB-0.5, MP3HB-1, MP3HB-2, and MP3HB-3, respectively. It can be seen that the weight loss temperature of all biocomposites is higher than that of unfilled P3HB. This dependence results from the higher decomposition temperature of HYP than of unfilled P3HB. The decomposition of HYP in N2 in accordance with the literature data [32] and manufacturer’s specifications equals onset at ~260 °C at 0.5 °C·min^−1^. The most desirable is the MP3HB-1 sample with the highest degradation temperature. Adding 1% filler by mass raises the degradation temperature of biocomposites by approximately 37 °C relative to unfilled P3HB.

**Figure 3:**
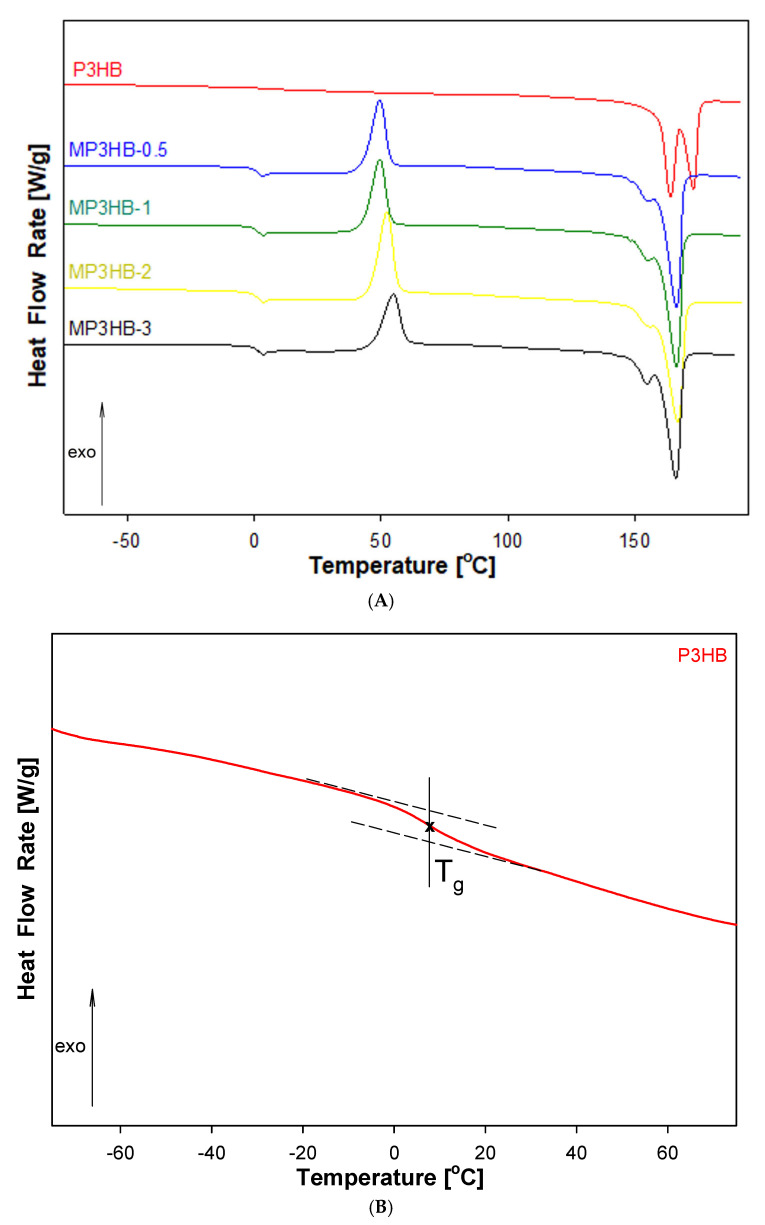
(**A**). Heat flow rate versus temperature, derived from DSC analysis of unfilled poly(3-hydroxybutyrate) and its biocomposites containing 0.5, 1, 2, and 3 mass % hypromellose. Samples are labeled MP3HB-0.5, MP3HB-1, MP3HB-2, and MP3HB-3, respectively. The data was gathered during heating at 10 °C/min, following cooling the samples at the same rate from the melt. (**B**). The enlarged glass transition region of the P3HB.

Figure 3 displays the heat flow rates of the prepared biocomposites (0.5, 1, 2, 3 wt.-%) as they vary with temperature, measured using standard differential scanning calorimetry (DSC).

The qualitative thermal analysis focused on assessing the heat flow rate of semicrystalline P3HB and its biocomposites. During the heating scan, the glass transition and melting were observed for all materials. All biocomposites showed cold crystallization as an exothermic peak within the range between the glass transition and melting temperatures (see Figure 3A). Thermal parameters of phase transitions are provided in Table 2. The glass transition temperature (T_g_) and the change in heat capacity at T_g_ (ΔC_p_) were determined from the heating of the glass transition region. Additionally, the heat of fusion (ΔH_f_) and the melting temperatures (T_m1_ and T_m2_) were estimated through analysis of the melting region. The inset (Figure 3B) highlights the enlarged area of the P3HB glass transition. The double melting peak remains visible, similar to the P3HB analysis. This is likely due to crystals with varying lamellae thicknesses [33].

The study investigated the impact of the HYP additive on the glass transition temperature (T_g_), melting temperature (T_m_), and the changes in heat of fusion and heat capacity at T_g_. Figure 3A shows the DSC curves for unfilled P3HB and its biocomposites with different hypromellose levels. Qualitative analysis of heat flow rate allowed for estimates of the change in heat capacity at T_g_ and the change in heat of fusion at T_m_ for both the unfilled semicrystalline P3HB and its biocomposites. Results show that the glass transition temperatures are lower than those of unfilled P3HB, indicating that hypromellose acts as a plasticiser within the P3HB matrix.

Our studies have explored blending P3HB with cellulose-derived polymers, including methylcellulose (HYP), as a strategy to overcome the inherent limitations of unfilled P3HB, such as brittleness and a narrow thermal processing window. Incorporation of HYP into P3HB matrices has been reported to improve thermal stability and overall structural integrity compared to unfilled P3HB. HYP, a water-soluble cellulose ether, has been investigated as a modifier for P3HB, providing enhanced flexibility and processability. In P3HB/HYP biocomposites, HYP can act as a plasticizing and compatibilizing component, mitigating the rigidity of P3HB and improving the hydrophilic properties of the composite.

The glass transition temperature of the biocomposite with the highest filler content increases by 7 °C compared to unfilled P3HB, and its melting temperature remains the same as the T_m_ of P3HB.

The notable and practical aspect is the noticeable distinction between degradation and melting temperatures, which aids processing and prevents material degradation. Notably, P3HB-1 exhibits the best thermal properties, with the widest processing window indicated by a 93.6 °C difference between these temperatures. This separation from melting and decomposition points in P3HB-based biocomposites enhances processability and safeguards the material. Additionally, the study analyzed heat of fusion and heat capacity variations in relation to the crystalline phase (W_c_), the flexible phase (W_a_), and the rigid amorphous phase (W_RAF_)—refer to Table 3.

**Figure 4:**
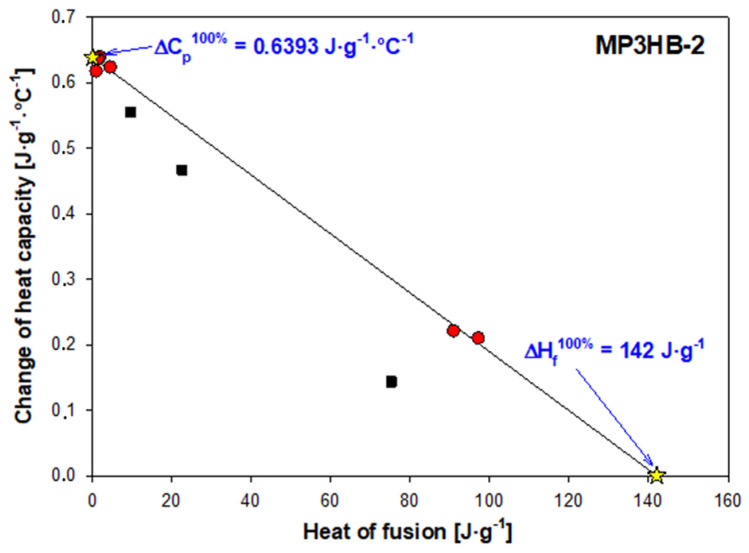
The relationship between the change in heat capacity at the glass transition temperature and the heat of fusion in semi-crystalline P3HB biocomposites with 2% hypromellose (labeled MP3HB-2). Red points indicate the two-phase biocomposite model, while black squares represent the three-phase model of MP3HB-2. The solid line connects the fully amorphous and fully crystalline MP3HB-2 samples, marked by yellow star points.

Figure 4 shows an example analysis of how the heat capacity at the glass transition temperature and the measured heat of fusion vary for semi-crystalline MP3HB-2 samples with different thermal histories after various cooling process rates. The glass transition of semi-crystalline MP3HB-2 varies from −0.20 °C to 15.60 °C depending on the cooling rate, with a ΔC_p_ (heat capacity change between liquid and solid at T_g_) ranging from 17.51 to 52.52 J∙mol^−1^·°C^−1^. The endotherm melting peak appears between 151.20 °C and 170.40 °C, with a heat of fusion between 78.77 and 8498.73 J∙mol^−1^. The change in heat capacity values was estimated from the qualitative thermal analysis, specifically from heat flow rate measurements. Red points denote a two-phase model, whereas black squares confirm a rigid amorphous fraction, indicating a three-phase system model. The solid straight line connects the fully amorphous and fully crystalline MP3HB-2 biocomposite states, marked by yellow star points. The heat capacity for fully amorphous MP3HB-2 is 55.95 J∙mol^−1^·°C ^−1^ (or 0.6393 J∙g^−1^·°C^−1^), and the change in heat of fusion for fully crystalline MP3HB-2 is 12.43 kJ∙mol^−1^ (or 142 J∙g^−1^).

**Figure 5:**
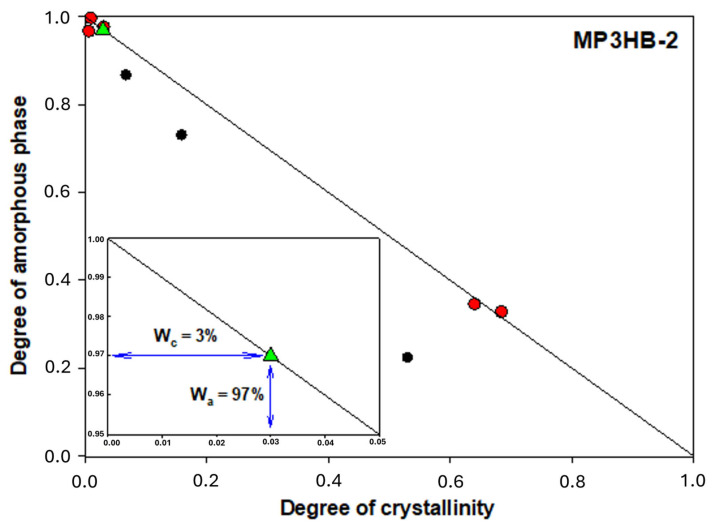
The amorphous fraction’s dependence on the crystallinity degree in the semi-crystalline poly(3-hydroxybutyrate) biocomposite with 2 wt.-% hypromellose, labeled as MP3HB-2.

Figure 5 illustrates the relationship between the mobile amorphous fraction and the degree of crystallinity in semi-crystalline MP3HB-2. Data from Figure 4 were recalculated to the mobile amorphous content using W_a_ = ΔC_p_/ΔC_p_^100%^, and the degree of crystallinity using the following equation: W_c_ = ΔH_f_/ΔH_f_^100%^. Here, W_c_ is the ratio of the sample’s heat of fusion (ΔH_f_) to that of fully crystalline MP3HB-2 (ΔH_f_^100%^). ΔC_p_ and ΔC_p_^100%^ are the heat capacity changes at T_g_ for semi-crystalline and fully amorphous MP3HB-2, respectively. The value used for ΔC_p_^100%^ is 55.96 J·mol^−1^·°C^−1^. Additionally, a green triangle point from qualitative analysis of MP3HB-2 is plotted in Figure 5, obtained after heating at 10 °C/min following cooling at the same rate from the melt. This point indicates that the sample remains within a two-phase model, confirmed by the linearity observed in Figure 5. The data show MP3HB-2 is composed of 97% mobile amorphous and 3% crystalline phases, with no evidence of a rigid amorphous phase (W_RAF_ = 0).

Similar analyses of other biocomposites were performed and are presented in Table 3. The same method used for MP3HB-2 was applied to determine the heat capacity change in amorphous materials and the heat of fusion in crystalline materials. In Table 3, phase contents are calculated for samples cooled at a controlled rate of 10 °C/min and subsequently heated at the same rate. These results suggest that adding hypromellose reduces the degree of crystallinity in the biocomposites.

**Figure 6:**
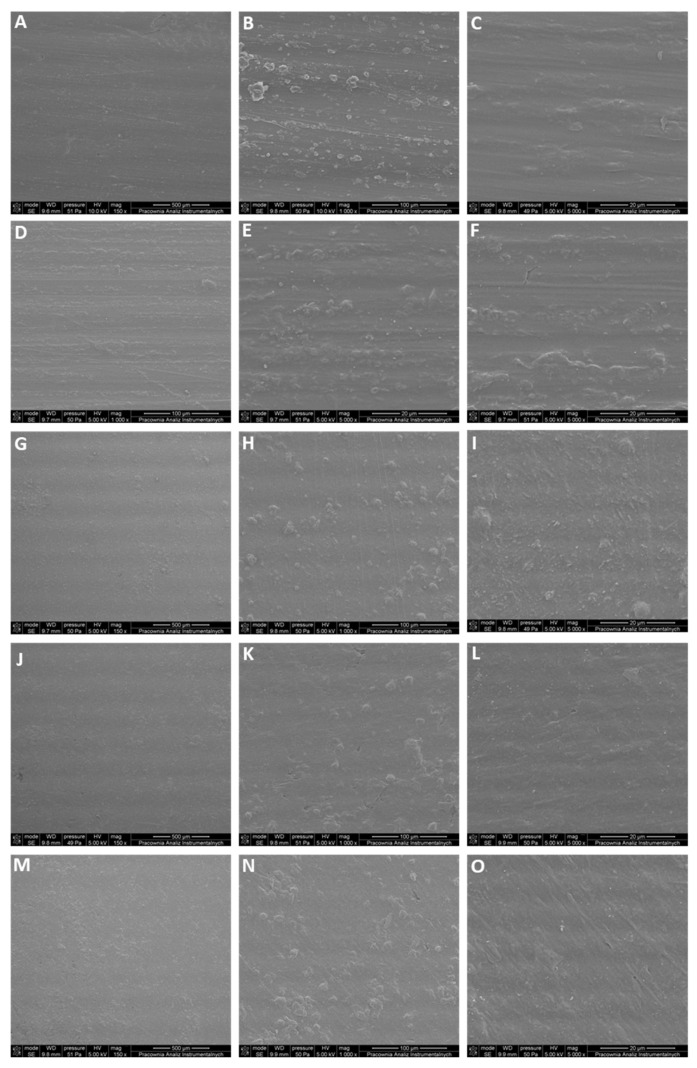
SEM micrographs of polymer surfaces: (**A**–**C**) P3HB; (**D**–**F**) MP3HB-0.5; (**G**–**I**) MP3HB-1; (**J**–**L**) MP3HB-2; (**M**–**O**) MP3HB-3.

The resulting micrographs based on SEM (Figure 6) were used to evaluate surface uniformity, filler dispersion, and other structural features. The unfilled P3HB (A–C) presents a compact, continuous surface with only faint, parallel processing marks. At higher magnification, the topography remains essentially featureless; no discernible pores or cracking are observed. This morphology is consistent with prior SEM studies of unfilled P3HB, which likewise report compact, continuous, and essentially featureless surfaces before any modification [34,35].

**Figure 7:**
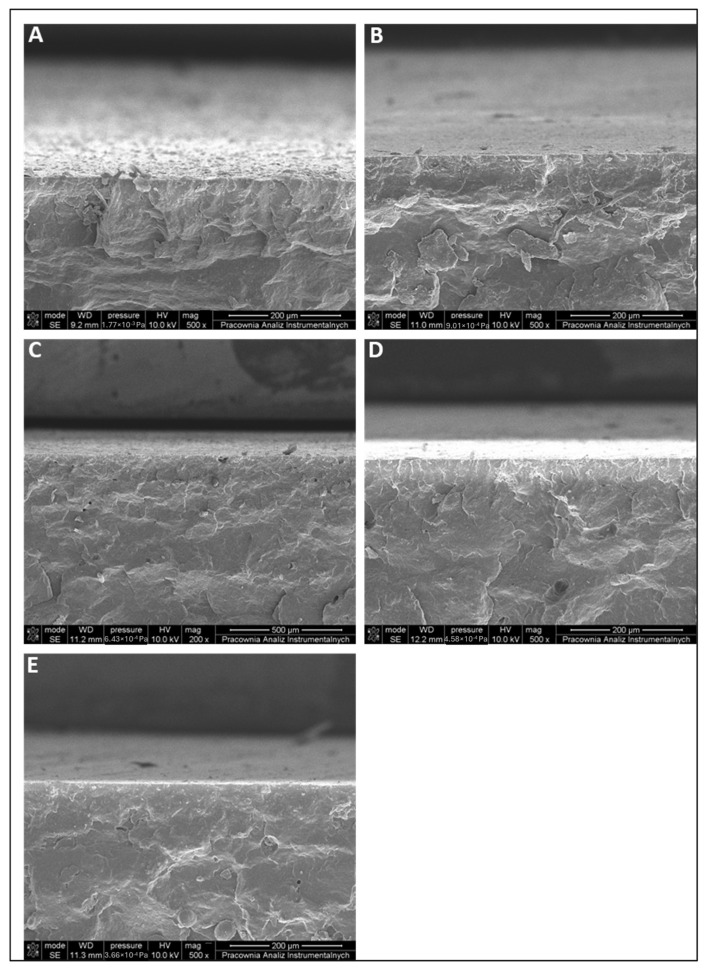
Cross-sectional SEM micrographs of (**A**) unfilled P3HB and P3HB/hypromellose (HYP) biocomposites: (**B**) MP3HB-0.5, (**C**) MP3HB-1, (**D**) MP3HB-2, and (**E**) MP3HB-3.

Introducing the modifier at the lowest levels (MP3HB-0.5; D–F and MP3HB-1; G–I) produces a surface that is still largely smooth, but sporadic sub-micrometric protrusions and rare shallow pits appear. In MP3HB-2 (J–L), numerous globular agglomerates populate the field and are accompanied by ridges and pits. At the highest loading (MP3HB-3; M–O), the surface shows a matte, uniformly roughened appearance already at low magnification. Dense, irregular agglomerates and scattered voids dominate the texture, and high-magnification views reveal oriented grooves and microcracking—hallmarks of phase separation and poor interfacial adhesion at elevated modifier content. Cross-sectional SEM assessment (Figure 7) reveals no substantial differences between neat P3HB and MP3HB composites containing 0.5–3.0 wt.-% hypromellose within the magnification and resolution employed. Despite the uniformly roughened surface seen for P3HB with 3 wt.-% hypromellose (MP3HB-3), SEM cross-sections remained dense and continuous, with no obvious voids or delamination. All specimens display a dense, continuous skin–core morphology with comparable brittle river/hackle markings; pores, pull-out cavities, and interfacial delamination are rare across the series. Observations clearly indicate that hypromellose was uniformly dispersed within the P3HB matrix.

Given the cellulose-based nature of hypromellose (C/H/O) and the absence of heavy elements, the compositional contrast between P3HB and hypromellose in SEM is minimal. Consequently, the hypromellose distribution cannot be directly distinguished in cross-sectional images; it is inferred indirectly from surface roughness, microporosity, and traces of local debonding. In the study by Seoane et al., nanocellulose (CNC) and bacterial cellulose (BC) are visible in SEM because they form more compact and stiff domains: CNC yields crystalline agglomerates with well-defined boundaries, whereas BC forms dense, continuous fibrous ribbons. This compact morphology generates strong topographic contrast (edges, steps, pull-out) and a fracture mode distinct from the PHB matrix, enabling identification of the cellulose phase despite similar (low) contrast. By contrast, hypromellose, as a water-soluble cellulose ether, does not form discrete compact grains; instead, it forms an interpenetrating phase that is often below the spatial resolution of SEM, explaining its limited visibility [36]. This is in line with the literature on PHB matrices modified with cellulose derivatives. For example, Seoane et al. reported that PHB films containing cellulose nanocrystals (CNC) exhibit good dispersion and smooth surface morphologies at low cellulose loadings, similar to our observations [36].

**Figure 8:**
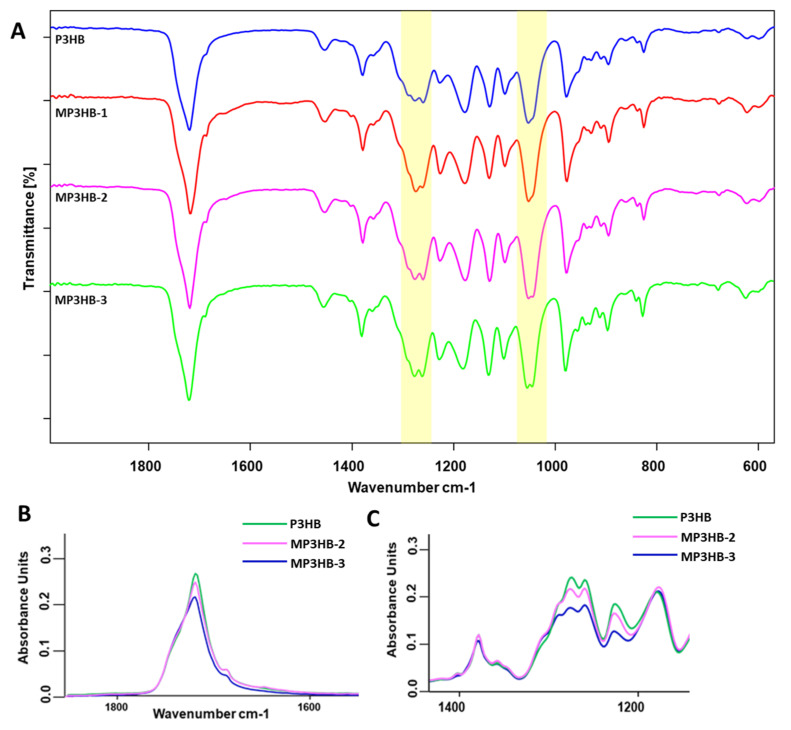
(**A**). ATR-FTIR spectra of P3HB (blue) and P3HB/hypromellose composites MP3HB-1 (red), MP3HB-2 (magenta), and MP3HB-3 (green); (**B**) amplified characteristic region of C=O; (**C**) C–O–C bonds.

The spectrum (Figure 8) of unfilled P3HB shows the characteristic bands present in all samples, including an intense ester carbonyl band at 1721 cm^−1^ and -C-O stretching bands at 1277 cm^−1^ and 1054 cm^−1^. Upon addition of hypromellose, a pronounced change appears in the fingerprint region: in MP3HB-2 and MP3HB-3, the single P3HB band evolves into a split (shouldered) feature centered near ~1053 cm^−1^. This component is plausibly attributed to the superposition of P3HB ν(C-O) vibrations with the carbohydrate C-O modes of hypromellose, whose contribution increases with additive loading.

Upon hypromellose addition, the 1250–1350 cm^−1^ band profile becomes noticeably narrower compared with neat P3HB. This window comprises the ν(C-O) modes of P3HB (~1270–1280 cm^−1^) together with a carbohydrate contribution from hypromellose around ~1310–1325 cm^−1^. The sharpening indicates a reduced distribution of local environments and conformational ordering of the backbone, consistent with HYP-P3HB hydrogen bonding; a modest nucleating effect of HYP may also contribute.

Figure 8B,C show a decrease in absorbance for the composites at ~1720 cm^−1^ (ν(C=O) of P3HB) and across the 1400–1150 cm^−1^ fingerprint region. The decrease in the carbonyl band upon hypromellose addition is consistent with intermolecular hydrogen bonding between P3HB carbonyls and hypromellose hydroxyls, in agreement with the TGA-indicated stabilization. Comparable FTIR changes, weakening/perturbation of the C=O band, together with modifications in the carbohydrate region, were likewise observed for PHB cellulose nanocomposites by Seoane et al. [36].

**Figure 9:**
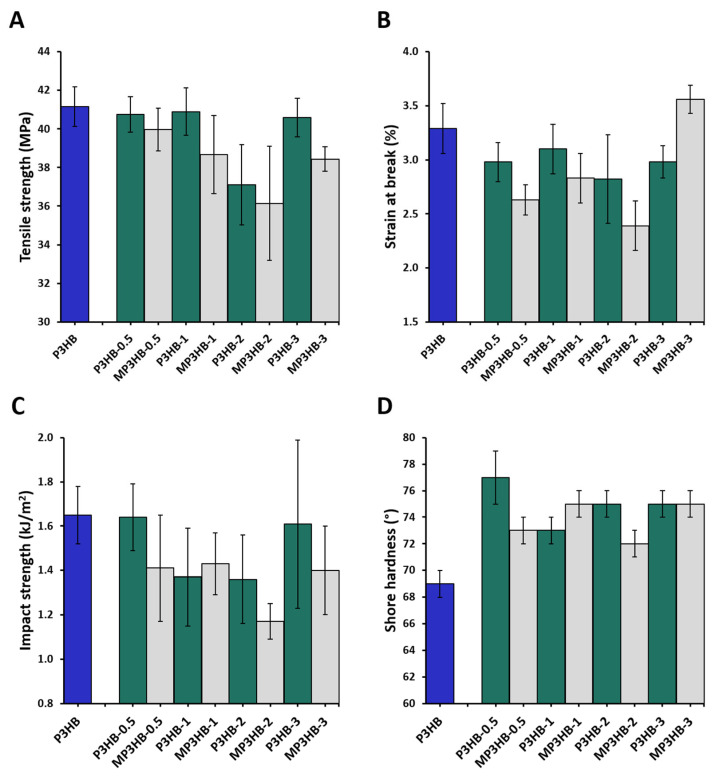
The content of hypromellose and nanocrystalline cellulose [37] influences (**A**) tensile strength, (**B**) strain at break, (**C**) impact strength, and (**D**) Shore hardness of poly(3-hydroxybutyrate) and its composites. These composites include varying amounts of additive—0.5, 1, 2, and 3 wt.-%—labeled as P3HB-0.5, P3HB-1, P3HB-2, P3HB-3 for nanocrystalline cellulose biocomposites [29], and MP3HB-0.5, MP3HB-1, MP3HB-2, MP3HB-3 for hypromellose biocomposites.

The study also examined how hypromellose affects the mechanical properties of P3HB-based biocomposites. For each sample, eight independent replicates of mechanical tests were conducted. The results were compared with earlier research involving nanocrystalline cellulose as a filler [37]. Figure 9 shows the test results as mean ± SD. A nonparametric Mann–Whitney U test was used for statistical analysis, with significance set at *p* < 0.05.

The introduction of hypromellose has a more significant impact on changing their mechanical properties compared to nanocomposites produced with unmodified nanocellulose. The results indicate a correlation between the content of the crystalline phase and RAF and the tensile strength. As the content of rigid domains decreases, the tensile strength decreases, reaching its lowest value for the MP3HB-2 biocomposite. Moreover, the lower content of hydroxyl groups present in hypromellose compared to nanocellulose results in limited interactions of the filler within the mobile amorphous phase of the polymer, among others, through the formation of hydrogen bonds, which consequently leads to lower tensile strength values compared to nanocomposites with unmodified nanocellulose. The tensile strength of the biocomposites was tested; it showed that hypromellose had a slight effect on it (Figure 9A). The results of the statistical analysis indicate significant differences were found only among M3HB-3 and P3HB-3 (*p* = 0.0038). P3HB differs significantly from MP3HB-1, MP3HB-2, and MP3HB-3. The statistical analysis shows significant differences between MP3HB-2 and all other biocomposites. The biggest difference is between P3HB-2 and P3HB (*p* = 0.015). The inclusion of hypromellose slightly reduced tensile strength overall, except when 2% methyl cellulose was added by weight, which caused a 12% decrease compared to unfilled P3HB.

Similarly to previous studies with nanocellulose, a reduction in strain at break was observed (Figure 9B). P3HB shows significant differences compared to all other biocomposites, with the most notable difference between P3HB and MP3HB-2 (*p* = 0.0008). This likely results from the presence of non-homogeneous crystalline domains. The formation of agglomerates can deteriorate the overall performance of the material. On the other hand, despite the increase in the mobile amorphous phase responsible for dissipating deformation energy, the biocomposites with hypromellose also exhibited lower impact strength and lower elongation at break. The results indicate that there is a statistically significant difference between P3HB-0.5 and MP3HB-0.5 (*p* = 0.0087), with the mean value for P3HB-0.5 being significantly higher. The difference between P3HB-3 and MP3HB-3 is also statistically significant. MP3HB-3 has a significantly higher mean value than P3HB-3 (*p* = 0.00016). This confirms the lower homogeneity of the biocomposite structure, in which the mobile amorphous phase is discontinuous and dispersed between the crystalline domains and RAF, which, by increasing stiffness and hardness, additionally act as crack-initiation sites, thereby limiting plastic deformation. However, in both cases, the use of 3 wt.-% of filler to P3HB resulted in an increase in the strain at break.

In the case of impact strength testing of the produced biocomposites (Figure 9C), it was observed that this parameter decreased statistically significantly compared to pure P3HB only when the hypromellose content was increased to 2% by weight (*p* = 0.004). Comparing P3HB-2 with MP3HB-2, we observe a decrease in impact strength of 18% and 30%, respectively, compared to P3HB. This shows that the use of hypromellose as a filler has a greater effect on impact strength than the addition of the same amount of nanocrystalline cellulose. These results indicate the stiffening effect of the filler.

According to the statistical analysis, the P3HB sample exhibited significantly lower values compared to all modified samples (Figure 9D). These results are consistent with the results of previous studies in which nanocellulose was used as a filler. An apparent 7% increase in the hardness of the produced biocomposites was observed, suggesting that the material’s structure became stiffer.

**Figure 10:**
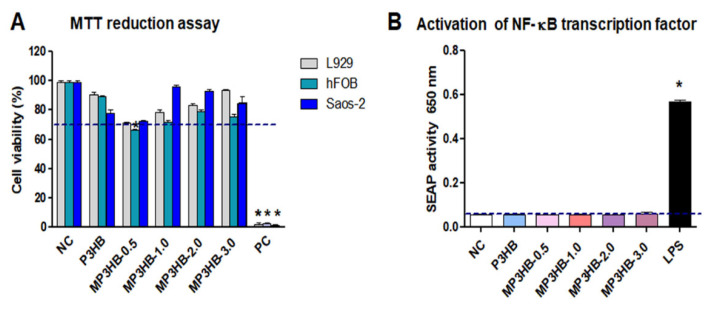
The biocompatibility and immunocompatibility of P3HB, MP3HB variants, and controls were assessed. Panel (**A**) shows cytotoxicity toward mouse L929 and human hFob 1.19 and Saos-2 cells, measured by MTT assay, with negative (H_2_O_2_) and positive controls. Results, obtained from three experiments conducted in triplicate, are expressed as mean ± SD, with a 70% (dashed lines) viability threshold indicating non-cytotoxicity; significance tested with the Mann–Whitney U test (*p* < 0.05). Panel (**B**) depicts SEAP activation in THP-1 ×Blue monocytes stimulated with LPS or P3HB variants, compared to controls. Supernatants were analyzed calorimetrically across three experiments in triplicate, and significance was determined using the Mann–Whitney U test (*p* < 0.05) (*: unstimulated versus stimulated cells).

To evaluate the potential applications of the newly developed biocomposites, assessments of cell viability and immunocompatibility within the tested material environment were conducted. The effects of P3HB, MP3HB-0.5, MP3HB-1, MP3HB-2, and MP3HB-3 on L929 mouse fibroblasts, human hFBO 1.19, and Saos-2 cells were assessed using the MTT assay. This assay measures mitochondrial dehydrogenase activity, with or without the substances. Mitochondrial enzyme activity correlates with cell viability. L929, hFOB 1.19, and Saos-2 cells treated with P3HB, MP3HB-1, MP3HB-2, and MP3HB-3 all show viability above 70%, indicating biological safety criteria (Figure 10). Therefore, these composites are suitable for contact with human tissue. Additionally, the L929 and Saos-2 cell lines maintained viability above 70% after exposure to MP3HB-0.5. However, the hFOB 1.19 cell line did not meet the safety threshold in vitro with the MP3HB-0.5 composite (Figure 10).

THP1-Blue™ cells, like other monocytes, are highly responsive to increased inflammatory reactions upon contact with foreign antigens. Their activation aims to detect and eliminate threats while restoring balance in the body. Overactivation—such as from biomaterial components or contaminants like endotoxins—can lead to tissue damage and breakdown of cellular barriers [38]. These cells have surface receptors, including TLR-2, TLR-4, TLR-5, TLR-6, and TLR-8, that interact with ligands like peptides and endotoxins. TLR stimulation activates NF-κB, leading to SEAP secretion into the media. In this study, we observed that THP-1xBlue monocytes exposed to LPS exhibited elevated SEAP levels, indicating NF-kappa B activation. The samples P3HB, MP3HB-0.5, MP3HB-1, MP3HB-2, and MP3HB-3 did not provoke SEAP activity beyond the negative control (cells in medium alone). This confirms the immunosafety of these extracts and the very low or absent endotoxin contamination. According to FDA and EMA guidelines, endotoxin levels in biomaterials contacting human blood should not exceed 0.25 EU. Our results showed that stimulating THP1-Blue™ monocytes with 0.25 EU endotoxin (positive control) increased SEAP production, with an absorbance of 1.5 ± 0.02 at 620 nm (Figure 10B).

**Figure 11:**
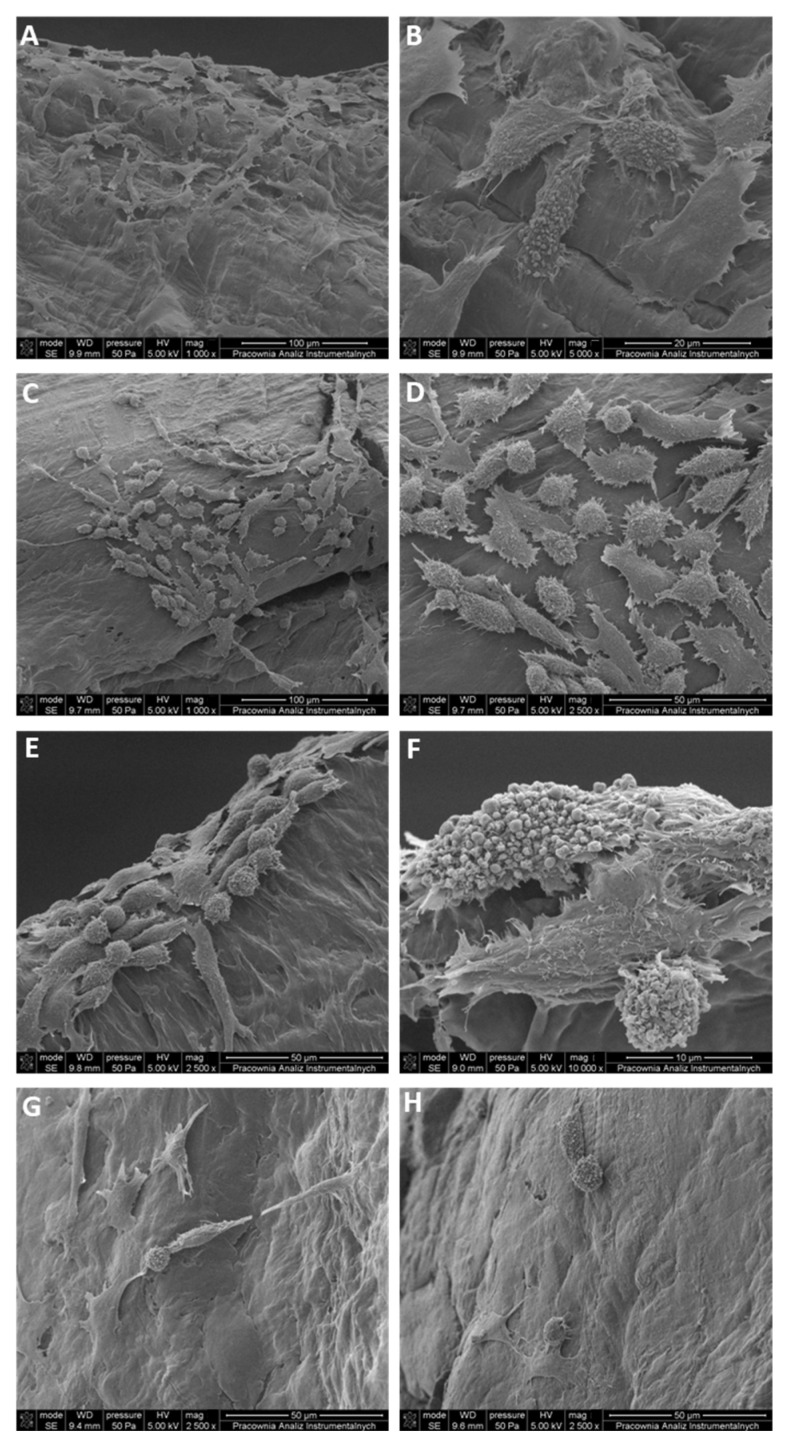
SEM images of L929 fibroblasts on (**A**,**B**) MP3HB-0.5, (**C**,**D**) MP3HB-1, (**E**,**F**) MP3HB-2, and (**G**,**H**) MP3HB-3. Scale bars are as indicated.

Scanning electron microscopy was used to analyze the surface topography of poly(3-hydroxybutyrate) (P3HB) and its biocomposites with varying amounts of hypromellose. On the unfilled P3HB (Figure 11A,B), L929 cells exhibit a typical fibroblastic phenotype with broad, flattened lamellae and multiple anchorage points. Cells are well spread over the continuous surface, showing numerous filopodial extensions that bridge shallow surface features; no signs of membrane damage are evident. On MP3HB-0.5 (Figure 11C,D), cell coverage is slightly lower, and spreading is limited relative to neat P3HB. Consistently, the MTT viability for this cell line was ~70%, i.e., at the typical threshold for non-cytotoxicity.

On MP3HB-1 (Figure 11E,F), cells are extensively spread, showing polygonal outlines with abundant filopodia and lamellipodia, consistent with strong attachment. However, on MP3HB-2 (Figure 11G,H), cell behavior is mixed; alongside elongated adherent cells, rounded cells and small clusters appear, indicating locally weaker adhesion.

Despite the near 100% MTT viability measured for MP3HB-3, SEM (Figure 11G,H) shows lower cell density and limited spreading, with slender cells bearing long, thin filopodia and occasional rounding, evidence of weakened cell–substrate interactions at the highest hypromellose content. By contrast, MP3HB-1 and MP3HB-2 achieved the most favorable balance, uniform coverage, broad spreading, and MTT viability > 70%, which indicates that intermediate hypromellose loadings support early cell anchorage while preserving biocompatibility; thus, these formulations are suitable for implantation for tissue reconstruction.

## 3. Materials and Methods

### 3.1. Materials

Poly(3-hydroxybutyrate), P3HB, was sourced from Biomer in Krailling, Germany. The melt flow index of P3HB is 0.11 g/10 min at 180 °C under a 2.16 kg load. Its weight-average molecular mass (M_w_) is 443,900 g/mol, with a molecular mass dispersion (M_w_/M_n_) of 5.72, measured via size exclusion chromatography in chloroform.

The filler used was hypromellose (methyl cellulose, HYP, CAS number: 9004-65-3), which was supplied by Sigma-Aldrich (Schnelldorf, Germany).

The molar masses of HYP and P3HB, with M_HYP_ = 478.49 g/mol and M_P3HB_ = 86.09 g/mol, respectively, were used to estimate the molar mass of the biocomposites: M_MP3HB-0.5_ = 85.53 g/mol, M_MP3HB-1_ = 86.80 g/mol, M_MP3HB-2_ = 87.52 g/mol, and M_MP3HB-3_ = 88.26 g/mol.

### 3.2. Methods and Instrumentation

#### 3.2.1. Biocomposites Preparation

The biocomposites were created through melt-mixing P3HB with different amounts of hypromellose—0.5, 1, 2, and 3 wt.-% —using a co-rotating twin-screw micro-extruder. This extruder featured four temperature-controlled zones: zone 1, zone 2, zone 3, and the feed zone. Prior to extrusion, the filler was homogenized with the polymeric matrix using an electric grinder (Fritsch GmbH, Idar-Oberstein, Germany). Table 4 provides the specific conditions for melt-mixing and comprehensive characterization of the P3HB and hypromellose-based biocomposites. Additionally, unfilled P3HB was melt-mixed to function as a reference material.

#### 3.2.2. Thermogravimetry

Thermogravimetric analysis (TGA) was performed using a Metler Toledo TGA/DSC 3+ (Mettler Toledo, Columbus, OH, USA); samples were heated at a rate of 5 °C per minute from 25 °C to 600 °C in a nitrogen atmosphere.

#### 3.2.3. Differential Scanning Calorimetry

All calorimetry experiments conducted between −90 °C and 195 °C utilized a Q1000TM DSC from TA Instruments, Inc. (New Castle, DE, USA). This heat-flux calorimeter features a mechanical refrigerator for both heating and cooling. Measurements were performed in a nitrogen atmosphere at approximately 50 mL/min, using samples with a mass of about 10 mg. The heat flow was recorded at a heating rate of 10 °C/min following a cooling process at the same rate, using standard DSC procedures. Calibration of temperature and heat flow rates was carried out with melting indium, set at an onset melting temperature of T_m(onset)_ = 156.6 °C and a heat of fusion of ΔH_f_ = 3.281 kJ/mol (28.45 J/g) [39]. Sapphire was used to calibrate the heat capacity to ensure the accuracy of measurements [39]. Data collection was from the second heating scan after controlled cooling.

#### 3.2.4. X-Ray Diffraction

X-ray diffraction methods (X-ray diffractometer Miniflex II from Rigaku company (Rigaku, Tokyo, Japan)) were applied to gain information about the structural changes and phases of the obtained composites. A CuKa (λ = 0.1542 nm) ray was employed for the analysis. The step size was configured to 0.02° over 3 s, with the applied voltage and current set at 40 kV and 10 mA, respectively. The diffraction angle (2θ) ranged from 3 to 30°.

#### 3.2.5. Mechanical Tests

The biocomposites and unfilled P3HB were tested for mechanical properties, including tensile strength (max force over cross-sectional area), elongation at break (percentage change in length), impact strength (energy to break divided by cross-sectional area using the Charpy method), and Shore hardness. Tests followed established standards: tensile and elongation per [PN-EN ISO 527-1:2020-01] [40], at 50 mm/min; impact using IMPats-15/50 hammer per [PN-EN ISO 179-1:2010] [41], Shore hardness on D scale with Zwick Roell equipment per [pn-ISO 868:1998] [42].

#### 3.2.6. Scanning Electron Microscopy

The surface morphology of unfilled P3HB and biocomposites containing 0.5, 1, 2, and 3 wt.-% hypromellose was examined using high-resolution scanning electron microscopy. To minimize charging and improve image quality, samples were sputter-coated with a gold/palladium (8:2) layer using a SC7620 sputter coater (Quorum Technologies, Laughton, UK). SEM images were captured with a Quanta 3D FEG (FEI, Hillsboro, OR, USA) at 10 kV.

##### Scanning Electron Microscopy of L929 Cells

For microscopic analysis, L929 cells at 2.5 × 10^4^ cells/mL were cultured on tested biocomposites at 37 °C with 5% CO_2_ for 2 days. Biocomposites were sterilized with ethanol and UV light before seeding. Adherent cells were fixed with 2.5% glutaraldehyde in PBS for 30 min, rinsed three times in PBS, then dehydrated in ethanol (95% for 2 min, then four 5 min steps in 100% ethanol). Cells were further dehydrated twice for 10 min in HMDS at room temperature. Polymeric samples with cells were mounted with carbon tape, gold-coated with a SC7620 Mini Sputter Coater (Quorum Technologies, Laughton, UK), and examined under a Quanta 3D FEG SEM/FIB (FEI, Hillsboro, OR, USA).

#### 3.2.7. ATR-FTIR Spectra

ATR-FTIR spectra were collected in the mid-IR region (4000–400 cm^−1^) for poly(3-hydroxybutyrate) (P3HB) and P3HB/hypromellose composites at various loadings using an Alpha FTIR spectrometer (Bruker, Billerica, MA, USA).

#### 3.2.8. Biological Studies

##### Cell Culture and In Vitro Biocompatibility Assessment

L929 mouse fibroblasts and human Hs68 skin fibroblasts were used to evaluate biocompatibility in vitro. Mouse L929 fibroblasts were cultured at 37 °C with 5% CO_2_ in RPMI-1640 medium (Biowest, Nuaillé, France), with 10% FBS and antibiotics: penicillin (100 U/mL) and streptomycin (100 µg/mL). Human fetal osteoblastic cells (hFOB 1.19), from ATCC, were grown in a 1:1 phenol-free DMEM and Ham’s 12-F medium (Gibco, Thermo Fisher Scientific, Grand Island, NY, USA), with L-glutamine, 10% FBS, and 0.3 mg/mL G418. Human osteosarcoma Saos-2 cells were cultured in McCoy’s 5A Medium with 15% FBS and antibiotics. Cultures were refreshed 2–3 times weekly and passaged with 0.25% trypsin-EDTA (Biowest). Cytotoxicity tests of P3HB, MP3HB-0.5, MP3HB-1, MP3HB-2, and MP3HB-3 used L929, hfob 1.19, and Saos-2 cells (2 × 10^5^ cells/mL) following ISO 10993–5 [43] and the MTT assay. Materials were prepared in 1/10 of the well surface. Control cells in medium served as positive, and cells with 0.03% H_2_O_2_ served as negative controls. Absorbance at 570 nm was measured using a Multiskan EX reader (Thermo Fisher Scientific Inc., Vantaa, Finland). MTT reduction was expressed as a percentage of untreated cells. All tests were performed in triplicate.

##### Monocyte Activation Assay

THP1-Blue™ cells from the human THP-1 monocyte line were used to determine if P3HB, MP3HB-0.5, MP3HB-1, MP3HB-2, and MP3HB-3 could activate monocytes by triggering NF-κb. These cells, equipped with an NF-κb-inducible SEAP reporter, produce SEAP upon NF-κb activation, which can be detected spectrophotometrically with Quanti-Blue. They were cultured at 37 °C in 5% CO_2_ in RPMI-1640 with 10% FBS, 25 mM HEPES, penicillin/streptomycin, 2 mM glutamine, and 10 μg/mL blastidin, then treated with the compounds. Untreated cells served as negative controls, while cells exposed to LPS (1 μg/mL) served as positive controls. SEAP activity was detected at 650 nm using a Multiskan EX plate reader. All experiments were conducted in triplicate, with three independent tests.

##### Statistical Analysis

Statistical significance was accepted at a *p*-value < 0.05 using the nonparametric Mann–Whitney U test. Data are presented as mean ± SD. For statistical analysis, the STATISTICA 12.3PL software was used (Statsoft, Krakow, Poland).

## 4. Conclusions

Biocomposites based on the P3HB and hypromellose were created by directly mixing them in a co-rotating twin-screw micro-extruder. The results clearly show that the filler in the P3HB matrix influences several properties of the biocomposites, such as their structure, thermal behavior, mechanical strength, and biological aspects.

Adding hypromellose improves the thermal stability of the biocomposites, as demonstrated by TG analysis under non-oxidative conditions. The highest thermal stability was noted in MP3HB-1, with a degradation temperature similar to the 2% weight loss point. Thermal analysis indicated a decrease in the glass transition temperature with increasing filler content, confirming the plasticising effect of hypromellose. An increased degradation temperature extends the processing window, making processing easier compared to unfilled P3HB. FTIR spectroscopy confirmed specific interactions between hypromellose and the P3HB matrix, with the shoulder band near 1053 cm^−1^ and a sharper region between 1250 and 1350 cm^−1^ indicating overlapping C-O vibrations and a more ordered P3HB backbone.

Adding hypromellose also reduced the crystallinity of the biocomposites and increased the amorphous phase, as validated by X-ray spectra and differential scanning calorimetry. Biological tests relevant to implant applications showed that the extracts are immunosafe and have minimal or no endotoxin contamination. In vitro studies further revealed that biocomposites MP3HB-1 and MP3HB-2 support cell attachment and spreading, which is promising for potential implant use.

## Figures and Tables

**Table 1:** The average crystallite size of biocomposites.

Sample	FWHM, °	D (nm)
P3HB	0.362	22.2
MP3HB-0.5	0.352	22.8
MP3HB-1	0.381	21.1
MP3HB-2	0.404	19.9
MP3HB-3	0.339	23.7

**Table 2:** Comparison of the thermal parameters for unfilled poly(3-hydroxybutyrate) and biocomposites with hypromellose was performed using representative samples heated and cooled at 10 °C/min.

Sample Designation	T_g_ [°C]	ΔC_p_ [J·mol^−1^·°C ^−1^]	T_m1_ [°C]	T_m2_ [°C]	ΔH_f_ [J/mol]
P3HB	7.70	15.22	–	165.80	8639.99
MP3HB-0.5	0.80	38.89	155.45	166.80	1877.31
MP3HB-1	0.60	44.70	155.20	166.50	34.72074
MP3HB-2	0.40	54.65	155.20	166.90	387.74
MP3HB-3	0.10	54.02	153.30	165.30	1006.18

**Table 3:** Characterization of phase contents in unfilled poly(3-hydroxybutyrate) and its biocomposites with hypromellose.

Sample Designation	Cp^100%^ [J·g^−1^·°C^−1^]	Cp^100%^ [J·mol^−1^·K^−1^]	Hf^100%^ [J·g^−1^]	Hf^100%^ [J·mol^−1^]	W_a_ [%]	W_c_ [%]	W_RAF_ [%]
P3HB	0.4900	42.18	145.0	12,483.05	33	63	4
MP3HB-0.5	0.6398	54.72	145.4	12,435.60	71.1	15.1	13.8
MP3HB-1	0.5770	50.08	147.9	12,837.99	89.3	0.3	10.4
MP3HB-2	0.6393	55.96	142.0	12,428.63	97	3	0
MP3HB-3	0.7345	64.83	120.4	10,626.68	83.3	9.5	7.2

**Table 4:** Sample abbreviations and preparation conditions of biocomposites and unfilled P3HB.

Sample Designation	Content of Hypromellose	Temperature of Extrusion	Speed of Rotation
P3HB	0	zone 1: 125 °C	350 rpm
MP3HB-0.5	0.5	zone 2: 135 °C	
MP3HB-1	1	zone 3: 150 °C	
MP3HB-2	2	zone 4: 160 °C	
MP3HB-3	3	head: 166 °C	

## Data Availability

The data will be made available on request.
